# Impact of oil type on the development and oral bioavailability of self-nanoemulsifying drug delivery systems containing simvastatin

**DOI:** 10.1038/s41598-024-71980-5

**Published:** 2024-09-29

**Authors:** Aya Hamdy, Mahmoud El-Badry, M. Fathy, Ahmed M. El-Sayed

**Affiliations:** Department of Pharmaceutics, Faculty of Pharmacy, Assiut University, Assiut, 71511 Egypt

**Keywords:** Essential oil, Ethyl oleate, SNEDDS, Pharmacodynamic study, Pharmacokinetic study, Biotechnology, Chemical biology, Nanoscience and technology

## Abstract

The aim of this work is to develop and evaluate self-nanoemulsifying drug delivery systems (SNEDDS) containing simvastatin to increase its oral bioavailability. Formulation EO 5 (Ethyl oleate 9.3% w/w: Tween 80 49.4% w/w: Propylene glycol 39.3% w/w) and Formulation CL 14 (Clove oil 54.3% w/w: Tween 80 34.4% w/w: Transcutol-P 9.3% w/w) were thoroughly studied. They showed emulsification time less than 1 min, droplet size in the nanometric range, and almost a complete drug release after 2 h. The in-vitro dissolution profile of both formulations was found to be significant in comparison to the pure drug in pH 1.2 and 7.4 buffers (*P* < 0.0001). Furthermore, they demonstrated superior anti-hyperlipidemic activity in comparison to simvastatin suspension (10 mg/kg/day). In order to investigate the impact of oil type on oral bioavailability, the selected formulations have been examined in terms of the in-vivo pharmacokinetic study, and formulation EO 5 was found to have higher bioavailability. After oral administration of a single dose (40 mg/kg) of simvastatin-loaded SNEDDS (CL14 and EO 5), a 1.5-fold and 1.95-fold increase in bioavailability were observed, respectively, as compared to simvastatin suspension. Hence, the results indicated that the developed SNEDDS could enhance the therapeutic efficacy and oral bioavailability of simvastatin.

## Introduction

Hyperlipidemia is defined as high levels of total cholesterol (TC) and non-high-density lipoprotein cholesterol (non-HDL-C) due to inadequate lipid metabolism^1^. Simvastatin (SIM) is frequently used to treat hypercholesterolemia, dyslipidemia, and coronary heart disease. Simvastatin inhibits the enzyme that converts 3-hydroxy-3-methyl-glutaryl-coenzyme A (HMG-CoA) into mevalonate, a precursor in the biosynthesis of cholesterol. Simvastatin is categorized as class II in the biopharmaceutical classification system because of its high lipophilicity (log *P* = 4.68) and low aqueous solubility (∼30 μg/mL)^2^. Simvastatin's slow rate of dissolution and extensive first-pass metabolism via cytochrome P3A (CYP3A) in the gut and liver result in a poor and variable bioavailability (5%) of the drug^3^. Various drug delivery systems, such as liposomes, cyclodextrin encapsulation, solid lipid nanoparticles, and nanostructured lipid carriers, have been developed to navigate around those constraints^4^. These approaches do, however, have some shortcomings. Solid lipid nanoparticles suffer from major limitations caused by lipid polymorphism, such as limited drug loading, the risk of gelation, and drug leakage during storage^2^. In the case of cyclodextrin, high concentrations (˃6 mM/L) cause hemolysis. Thermodynamic instability in liposomes can result in aggregation, hydrolysis, or degradation, ultimately causing the leakage of drugs. Self-nanoemulsifying drug delivery system (SNEDDS) is widely recognized for its ability to overcome limitations related to poorly bioavailable drugs by using a variety of mechanisms, such as increasing membrane fluidity and permeability to facilitate transcellular transport, opening tight junctions to allow paracellular transport, and inhibiting P-gp and/or cytochrome P450 enzymes to increase drug concentration. Furthermore, the specific components of SNEDDS may avoid the first-pass effect of some drugs by promoting their intestinal lymphatic transport^5^. Long-chain fatty acid (LCFA)-based lipids considerably improved the lymphatic transport of the novel antimalarial drug N-251 (logP, 6.67), resulting in a significant enhancement of bioavailability^6^. Furthermore, the research conducted by Yamanouchi has shown that lipoidal components, specifically LCFA-based lipids, more effectively enhanced the lymphatic transport of clofazimine than did MCFA-based lipids^7^. SNEDDS are isotropic mixtures of oil, surfactant, and co-surfactant that form an oil-in-water nano- or microemulsion upon mild agitation followed by dilution in aqueous media^8^. SNEDDS has several advantages, including improved thermodynamic stability, fill-in unit dosage forms, large-scale manufacturing, and patient compliance.

The current study aims to develop simvastatin-loaded SNEDDS comprised of two distinct types of oils: essential oils and long-chain fatty acids, in order to examine the impact of oil type on the system's physicochemical characteristics and bioavailability. Furthermore, because scientific articles frequently use nano- and micro-emulsions arbitrarily, the study closely evaluates the nature of dispersions that emerge after self-emulsification.

## Materials and methods

### Materials

Simvastatin was a kind gift from PHARCO Pharmaceuticals Inc. (Alexandria, Egypt). Clove oil (pure, assay 99% min) and cinnamon oil (pure, assay 98% min) were obtained from Alpha Chem Co. (Cairo, Egypt). Labrasol® (caprylocaproyl polyoxyl-8 glycerides) and Transcutol-P (diethylene glycol monoethyl ether) were kindly donated by Gattefosse CO. (Saint Priest, France). Cremophor RH40® (polyoxy 40 hydrogenated castor oil) was a gift sample from BASF (Ludwigshafen, Germany). Tween 80, Tween 20, and propylene glycol were obtained from El-Nasr Pharmaceutical Chemicals Co. (Cairo, Egypt). All other chemicals used were of analytical grade and used as received.

### Solubility study

Simvastatin's solubility was assessed in various vehicles utilizing a previously described method^9^. Two grams of each vehicle were combined with an excess amount of simvastatin, and the resulting mixtures were vortexed for three minutes in order to aid in the solubilization of the drug. The mixtures were maintained at 37 ± 0.5 °C for 72 h at 100 rpm in a temperature-controlled shaking water bath (DAIHAN Scientific Co. Ltd., Type WSB-30, Korea)^10^. The undissolved drug was subsequently extracted from the samples by centrifuging them for 15 min at 10,000 rpm. A 0.45 μm membrane filter was used to filter the supernatant solution. An Evolution 300 BB double beam UV–vis spectrophotometer (Thermo Scientific, England) was utilized to examine all solutions spectrophotometrically at λ_max_ of 238 nm. The dilution ratio of each solution was the same as that of the pure vehicle under study in methanol used as a blank^8^.

### Preliminary screening of surfactants and co-surfactants

The ability of various surfactants to emulsify the chosen oil phases was assessed using the method described by Date and Nagarsenker^11^. After mixing equal parts oil and surfactant, the mixture was vortexed for two minutes to ensure all the components were homogenized. 50 mg were carefully weighed and diluted to 50 ml with distilled water in order to form SNEDDS. The ease of emulsification was determined by counting the number of flask inversions. After two hours, each produced emulsion was visually inspected for turbidity, and the percentage transmittance (T%) was measured using an Evolution 300 BB double beam UV–Vis spectrophotometer at 638.2 nm^10^. In order to examine the co-surfactants' emulsification capability, 200 mg of surfactant, 100 mg of co-surfactant, and 300 mg of oil were thoroughly mixed and assessed in accordance with the procedure previously described^12^.

### The nature of obtained dispersions

By examining the effect of temperature changes and the order of mixing of SNEDDS excipients, the nature of the resulting dispersions was investigated. After diluting plain SNEDDS 1000 times with distilled water, the clear dispersions were stored at − 10, 4, 25, and 40 °C consecutively for 24 h at each temperature^13^. The impact of temperature variations on the different dispersions was assessed visually. In order to examine the impact of mixing order, plain SNEDDS were reconstituted with the same oil/surfactant/co-surfactant ratios but with a different mixing order^14^. Prior to adding the oil phase, the surfactant/co-surfactant mixture was first dissolved in distilled water. The dispersions produced by dilution by a factor of 1000 were assessed visually.

### Construction of pseudo-ternary phase diagrams

In light of the drug's solubility and emulsification capability results, cinnamon oil, ethyl oleate, and clove oil were chosen as oily phases; tween 80 was chosen as a surfactant; and labrasol, propylene glycol, and transcutol-P were chosen as co-surfactants to construct three pseudo-ternary phase diagrams. For each phase diagram, several formulations were developed using various concentrations of oil, surfactant, and co-surfactant^12^. In order to assess the formulations' capability for self-emulsification, 50 mg of each formulation were diluted to 50 ml with distilled water, and after two hours, the percentage transmittance was determined^11^. Formulations of grade A, which produced clear dispersions with a percentage transmittance of over or equal to 95%, and grade B, which formed translucent dispersions with a percentage transmittance of over or equal to 90%, were accommodated in the nano-emulsion region of the diagram^15^. The three-phase diagrams were generated using CHEMIX ternary plot software (CHEMIX School Ver. 3.60, Pub. Arne Standnes).

### Preparation of simvastatin-loaded SNEDDS

Thirty SNEDDS formulations in the self-emulsifying areas were chosen for drug incorporation and additional characterization (Supplementary Table S2). A set dose of simvastatin (10 mg) was dissolved in various ratios of oil, surfactant, and co-surfactant to develop simvastatin-loaded SNEDDS formulations. To guarantee uniform drug distribution, the mixture was subsequently mixed using a vortex mixer.

### Stability of SNEDDS preconcentrates

To evaluate the stability of simvastatin-loaded SNEDDS, formulations were subjected to three distinct tests, including centrifugation, heating–cooling, and freeze-thawing. Firstly, in the centrifugation study, the chosen SNEDDS formulations were diluted 1:25 with distilled water, centrifuged for 30 min at 5000 rpm, and then visually inspected for drug precipitation, or creaming^16^. Formulations that passed were chosen for heating–cooling cycles, undergoing six cycles at 4 and 40 °C, with 48 h of storage at each temperature. After being diluted with distilled water (1:25), formulations that did not exhibit any evidence of instability were subjected to freeze–thaw cycles^17^. The formulations underwent three cycles of freezing and thawing at − 20 °C and 25 °C. They were stored at each temperature for 48 h before being centrifuged for five minutes at 3000 rpm. The formulations were then diluted with distilled water (1:25) and checked for indications of instability^17^.

### Robustness to dilution

To simulate conditions encountered when delivered orally, each formulation was diluted 10, 100, and 1000 times using 0.1 N HCl, phosphate buffer (PH 6.8), and distilled water^10^. After two hours, the formulations were visually evaluated. They were then kept for 24 h before being examined for cloudiness, phase separation, or precipitation^9^. Passed formulations yielded clear or slightly bluish dispersions at various dilution folds in either diluent.

### Assessment of self-emulsification efficiency

In order to assess the self-emulsification time, 1 ml of each formulation was dispersed into 250 ml of phosphate buffer (pH 6.8), 0.1 N HCl, or distilled water in a standard USP 24 dissolution apparatus II (Erweka, DT-D6, Heusenstamm, Germany)^14^. A moderate stirring was achieved by setting the paddle speed to 50 rpm while keeping the temperature constant at 37 ± 0.5 °C. The time needed to generate a homogeneous and emulsified dispersion was assessed^10^. Using the five grading systems, the self-emulsification efficiency of different formulations was assessed visually (Table 1)^18^.

**Table 1:** Grading system for evaluating self-emulsification efficiency.

Observation	Time of self-emulsification	Grade
Rapid forming emulsion which is clear or slightly bluish in appearance	Within 1 min	A
Rapid forming, slightly less clear emulsion which has a bluish white appearance	Within 2 min	B
Bright white emulsion similar to milk	Within 3 min	C
Slow forming, dull, grayish white emulsion with a slightly oily appearance	Longer than 3 min	D

### SNEDDS characterization

Based on the results of robustness to dilution and self-emulsification efficiency, nine formulations were selected for further characterization.

#### Cloud point measurement

In order to evaluate the effect of temperature on the stability of the resulting dispersions, the cloud point is an important factor. Formulations were diluted at a ratio of 1:100 with distilled water and placed in a water bath with a gradual increase in temperature^12^. The cloud point was distinguished as the temperature at which the formulation became cloudy^9^.

#### Droplet size, polydispersity index and zeta potential determination

Zeta potential, mean droplet size, and polydispersity index of nanoemulsions obtained after 100-fold dilution of selected formulations with distilled water were determined by the dynamic light scattering (DLS) technique using the Malvern Zetasizer Nano Series ZS (Malvern Instruments, UK)^9^.

#### In-vitro drug release

The dissolution studies with simvastatin were conducted under sink conditions using the USP apparatus II paddle method^9^. Samples of SNEDDS and drug suspension containing 4 mg of simvastatin were added to 900 mL of dissolution medium (pH 7.4) and (pH 1.2) at 37 °C. Simvastatin was added to distilled water containing 0.5% w/v sodium carboxymethyl cellulose (CMC) to prepare the drug suspension. Five milliliters of each vessel's contents were removed and replaced with an equal volume of fresh medium at predefined intervals (10, 20, 30, 40, 50, 60, 80, 100, and 120 min). Simvastatin's concentration was measured using a UV spectrophotometer at 238 nm. All measurements were carried out in triplicate. Several kinetic models, including zero-order, first-order, Higuchi, and Korsemeyer-Peppas models, were applied to study the drug release mechanism of the SNEDDS formulations.

### Further in-vitro characterization of the selected formulations

#### Transmission electron microscopy (TEM)

Transmission electron microscopy (TEM) was used to analyze the morphology and structure of the chosen formulations, CL14 and EO5, 24 h after a 1000-fold dilution with distilled water using the TECNAI G2 Spirit Twin (FEI, USA). On a copper grid, one drop of the sample was placed, and it was left to dry^19^. The grid was stained using a 2% w/v solution of phosphotungstic acid^14^. Before evaluation, extra phosphotungstic acid was eliminated by absorbing it on filter paper and allowing it to dry for five minutes.

#### Stability study for selected simvastatin-loaded SNEDDS formulations

Selected SNEDDS formulations were stored for 3 months at room temperature (25 °C) and evaluated for optical clarity, droplet size, zeta potential, emulsification time, and drug content^10^.

### In-vivo anti-hyperlipidemic activity of simvastatin-loaded SNEDDS formulations

#### Hyperlipidemia induction and treatment with simvastatin-loaded SNEDDS

The anti-cholesterolemic effects of different SNEDDS were compared with simvastatin suspension using a poloxamer-induced hyperlipidemia rat model^20^. It is well known that the non-ionic surfactant poloxamer 407 can induce hyperlipidemia quickly after a single intraperitoneal injection^20^. The study involved the random assignment of thirty male albino rats (300 ± 50 g) into five groups, each consisting of six animals: group I was the control group, which received normal saline; group II was the hyperlipidemic control group, which received normal saline; group III received oral simvastatin suspension; and groups IV and V received oral treatment with specific SNEDDS formulations, CL 14 and EO 5, respectively. To produce the drug suspension, 10 mg of simvastatin were suspended in 10 ml of distilled water containing 0.5% w/v sodium carboxymethyl cellulose (CMC)^4^. The rats were fasted overnight prior to the study, with free access to water. Poloxamer 407 solution (20% w/v) was administered intraperitoneally at a dose of 1 g/kg to induce hyperlipidemia^4^. After 12 h of receiving a poloxamer injection, the rats were given numerous oral doses of drug suspension and formulations over three days (dosage = 10 mg/kg/day)^21^. After injecting poloxamer (or normal saline) into each group of rats, blood samples were taken from the retro-orbital sinus at 0, 12, 36, and 60 h. Blood samples were put in clot activator tubes, allowed to stand at room temperature for half an hour, and then centrifuged for ten minutes at 10,000 rpm in order to separate the serum. They were then kept for additional analysis at − 20 ºC.

#### Biochemical analysis

The blood samples were analyzed to obtain baseline values of total cholesterol (TC) and high-density lipoproteins (HDL). The non-high-density lipoprotein cholesterol (non-HDL-C) was determined by using Eq. (1).

1 $$ {\text{Non}}\;{\text{HDL}} - {\text{C}} = {\text{Total}}\;{\text{cholesterol}} - {\text{HDL}} - {\text{C}} $$

Both the percentage of initial TC level and the percentage of initial non-HDL-C level were calculated using Eqs. (2 and 3):

2 $$ \% \;{\text{of}}\;{\text{initial}}\;{\text{TC}}\;{\text{level }} = {\text{measured}}\;{\text{TC}}\;{\text{level}}/{\text{baseline}}\;{\text{TC}}\;{\text{level}} \times 100 $$

3 $$ \begin{gathered} \% \;{\text{of}}\;{\text{initial}}\;{\text{non}} - {\text{HDL}}\;{\text{level}} = {\text{ measured}}\;{\text{non}} - {\text{HDL}} - {\text{C}}\;{\text{level}}/{\text{baseline}}\;{\text{non}} \hfill \\ - {\text{HDL}} - {\text{C}}\;{\text{level}} \times {1}00 \hfill \\ \end{gathered} $$

### Pharmacokinetic study

A pharmacokinetic study was performed to investigate the ability of SNEDDS to improve simvastatin oral bioavailability. The rats were randomly divided into three groups (three animals per group) and received a single oral dose of simvastatin (40 mg/kg) in the form of free drug suspension for the first group, CL 14 formulation for the second group, and EO 5 for the third group. Although this dose is higher than the clinically used dose, pharmacokinetic studies in rodents suggest that greater statin doses (in comparison to human doses) are necessary to get comparable efficacious concentrations^22^. In order to determine low quantities of the medication in the plasma (on the nanogram scale), a greater dose (40 mg/kg) was utilized in the pharmacokinetic tests compared to the pharmacodynamic studies (10 mg/kg). Prior to receiving the formulations, all rats were fasted for the whole night. Four hours after the treatments, they were fed again. At the predetermined intervals (1, 2, 4, 8, 12, and 24 h after administration), 0.5 mL of blood were drawn via vein puncture from the caudal vein and placed into heparinized tubes. After being separated by centrifugation at 3000 rpm for 10 min, the plasma samples were frozen at − 20 °C for additional analysis. The simvastatin concentrations in plasma were analyzed using a reversed-phase HPLC as described previously, with some modifications^23^. The HPLC method was performed on a Dionex Ultimat 3000 UHPLC system (Thermo Scientific, Waltham, MA) equipped with a HPG-3200 RS pump, a DAD-3000 RS detector, a WPS-3000TRS analytical autosampler, and a Hypersil BDS C18 analytical column (dimensions of 150 mm × 4.6 mm ID × 5 µm). The mobile phase is composed of acetonitrile and deionized water (65:35 *v*/v) and was adjusted to pH 3.5 by phosphoric acid^4^. The flow rate was set at 1.0 mL/min, the injection volume was 20 µL, and the elute was analyzed with a DAD detector set at a wavelength of 238 nm. The procedure used to prepare the plasma samples was to add 1 mL of acetonitrile to 200 µL of plasma to precipitate the plasma proteins^24^. The samples were then vortexed and centrifuged for 20 min at 13,000 rpm. After being collected, the supernatant was subjected to HPLC analysis after being filtered using a 0.45 µm syringe filter (Millipore, Billerica, MA). Blank plasma spiked with standard simvastatin solutions was used to create a calibration curve of the drug's concentration in plasma, obtaining a concentration range of 0.01–5 µg/mL. After that, the samples of spiked plasma underwent the same extraction process as the other samples.

#### Pharmacokinetics analysis

The pharmacokinetic parameters were calculated using the PK Solver 2.0 software. The area under the plasma concentration–time curve from zero to infinity (AUC_0-∞_) was calculated using the trapezoidal rule method. The maximum concentration (C_max_), time to reach the maximum concentration (T_max_), elimination half-life (t_½_) and mean residence time (MRT) were calculated. The relative bioavailability (F%) of formulations after oral administration was calculated according to Eq. 4:

4 $$ \begin{gathered} {\text{Relative}}\;{\text{bioavailability}}\left( {{\text{F}}\% } \right) = \left( {{\text{AUC}}} \right){\text{simvastatin}} \hfill \\ - {\text{loaded}}\;{\text{SNEDDS}}/\left( {{\text{AUC}}} \right){\text{simvastatin}}\;{\text{suspension}} \times {1}00 \hfill \\ \end{gathered} $$

### Ethics declarations

- The study protocol and animal care procedures were reviewed and approved by the Institutional Animal Ethical Committee of the Faculty of Pharmacy, Assiut University, and maximum efforts were made to minimize animal suffering (Approval No. S19-21). This committee is a branch of the licensed and approved Assiut University Research Ethics Committee (AUREC). According to standard procedures, the candidate requests permission from the local faculty ethical committee to conduct research on humans or animals. A copy of the original study protocol sheet, which outlines the planned procedures for either humans or animals as well as the goal of the investigation and its significance, is attached to his request. The committee schedules a time to speak with the candidate face-to-face about the issue and asks any questions that need to be addressed. After that, the candidate received the approval sheet.
- All procedures followed Chapter 4 of the official Assiut University Research Ethics Committee (AUREC) guidance.
- ARRIVE guidelines were also reviewed, and all methods were implemented in compliance with the guidelines' ten comments regarding the use of animals in research.

### Statistical analysis

Statistical analysis was carried out using GraphPad Prism version 7.01 for Windows, GraphPad Software, La Jolla, California, USA. A one-way ANOVA followed by a Tukey post hoc test was performed to determine statistically significant differences between different formulations. The differences were considered to be significant at *P* ≤ 0.05.

## Results and discussion

### Solubility study

**Fig. 1:**
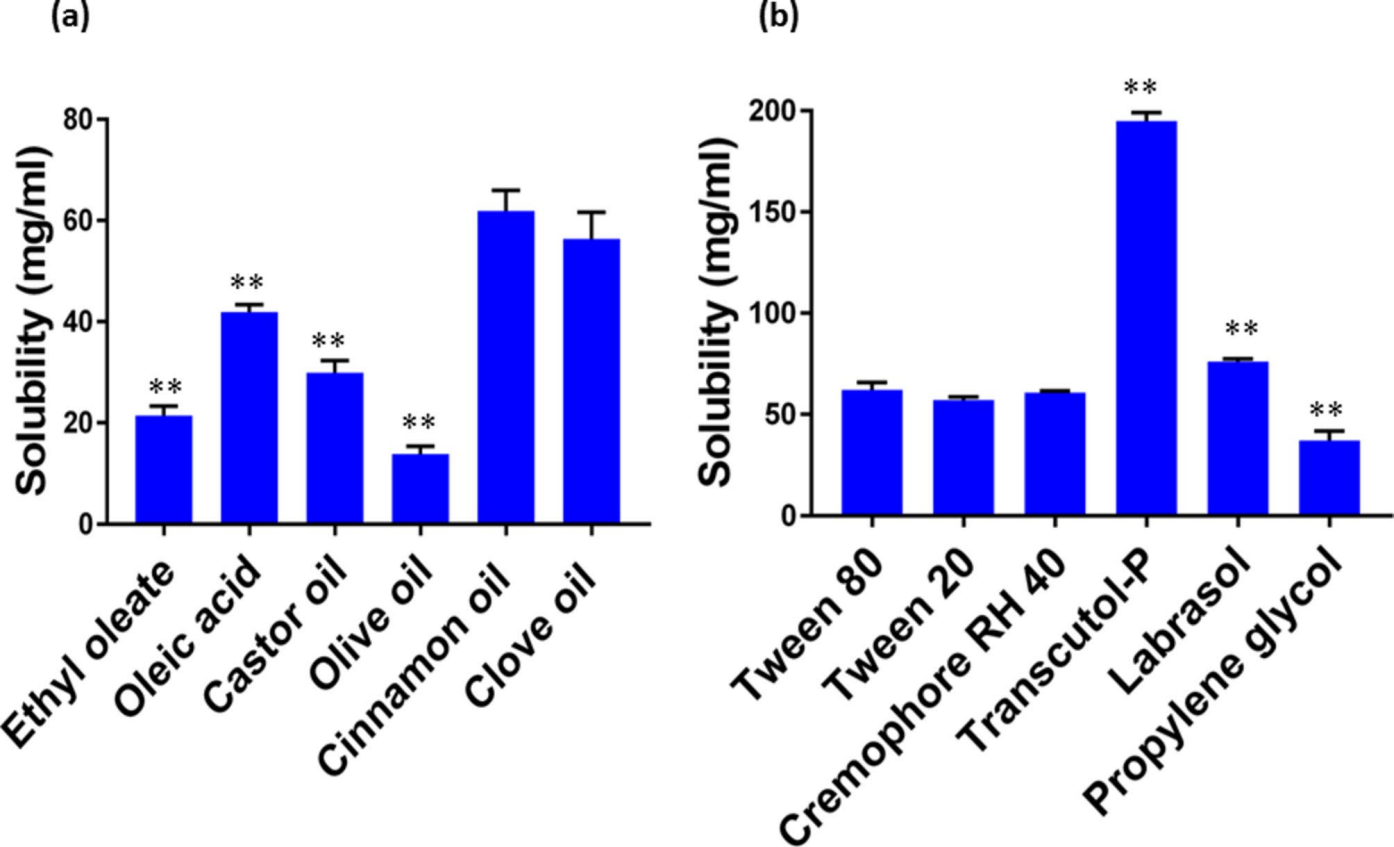
(**a**) Solubility of simvastatin in various oils; *P < 0.05 when compared to cinnamon oil; **P < 0.001 when compared to cinnamon oil; (**b**) solubility of simvastatin in surfactants and co-surfactants. *P < 0.05 when compared to tween 80; **P < 0.001 when compared tween 80; data presented as mean ± SD, n = 3.

To avoid drug precipitation upon dilution in the gut, optimum selection of SNEDDS components to achieve the highest drug loading is an important factor^15^. Simvastatin solubilization capacity was used as the basis for choosing the oil phases to ensure lower amounts of surfactant and co-surfactant, thus minimizing their toxic effects^25^. The solubility of simvastatin in both cinnamon oil (61.93 ± 4.04 mg/mL) and clove oil (56.356 ± 5.26 mg/mL) was significantly superior to that in other oils screened (*P* < 0.001), thus they were selected as the oil phases (Fig. 1). In the present SNEDDS formulation, ethyl oleate (21.45 ± 1.83 mg/mL**)** was also chosen as the oil phase due to its capacity to stimulate the intestinal lymphatic pathway. Hence, drugs can avoid the hepatic first-pass metabolism^26^. Cremophor RH 40, Tween 20, and Tween 80 were evaluated for surfactant selection, while Labrasol, Transcutol-P, and propylene glycol were evaluated for co-surfactant selection. There was no significant difference in the solubility of all the screened surfactants (*P* = 0.085) (Fig. 1). Among the screened co-surfactants, the maximum solubility for simvastatin was seen with Transcutol-P (194.92 ± 4.09 mg/mL), followed by Labrasol (76.09 ± 1.4 mg/mL).

### Preliminary screening of surfactants and co-surfactants

Because they are less hazardous and less impacted by changes in pH and ionic strength, non-ionic surfactants are recommended for oral consumption over ionic surfactants^10,12^. Furthermore, it has been demonstrated that non-ionic surfactants such as Span, Tween, and Cremophor inhibit CYP3A metabolism or P-glycoprotein drug efflux, thus improving the medication's intestinal absorption^27^. Both cinnamon and clove oils demonstrated the highest emulsification efficiency with all tested surfactants when compared to other oils (Table 2). A single flask inversion (one second) was sufficient for both oils to form a homogenous emulsion. Since the amount of oil used in the emulsion and its emulsification depend on the oil's molecular volume, ethyl oleate demonstrated poorer emulsification ability than essential oils^28^. By increasing the number and length of hydrophobic alkyl chains, the molecular volume increases, thus reducing the emulsification ability of the oil. The primary criterion taken into consideration while choosing surfactants is their ability to emulsify^29^. For both clove and cinnamon oils, Tween 20, Cremophor RH40, and Tween 80 showed outstanding and comparable emulsification abilities. Tween 80 was selected for SNEDDS formulations due to its emulsification ability and lymphotrobic character. Co-surfactants are crucial in the formulation of SNEDDS because they enter the interphase and create some void spaces for water to enter, which causes spontaneous emulsification due to enhanced interfacial fluidity^30^. Moreover, they form a mechanical barrier against coalescence, thus enhancing the resulting nanoemulsion stability^17^. Regarding essential oil-based systems, there was a non-significant difference between the emulsification efficiency of the screened co-surfactants (Table 3). However, the results of the ethyl oleate-based system showed that the emulsification efficiency of propylene glycol was significantly higher than that of labrasol or transcutol-P (*P* = 0.0001, *P* = 0.0041, respectively). Transcutol-P was used in the clove oil-based system as it modifies the film curvature, which promotes drug loading into the SNEDDS and self-dispersibility properties. Due to its ability to open tight connections in the intestinal membrane and promote intestinal absorption, Labrasol was employed in the cinnamon oil-based system^31^.

**Table 2:** Emulsification efficiency of various surfactants using different oil phases; data are expressed as the mean value ± S.D. (n = 3).

% Transmittance			
Oil	Tween 20	Tween 80	Cremophore RH 40
Cinnamon oil	100.69 ± 0.46	100.54 ± 0.71	101.08 ± 0.38
Clove oil	100.31 ± 0.35	100.69 ± 0.61	100.23 ± 0.23
Oleic acid	27.51 ± 2.1	35.42 ± 2.2	46.25 ± 2.67
Ethyl oleate	89.15 ± 2.5	29.08 ± 1.95	75.76 ± 1.84
Olive oil	61.72 ± 1.42	41.44 ± 1.098	19.68 ± 1.37
Castor oil	64.72 ± 0.98	46.92 ± 5.18	84.4 ± 1.75

**Table 3:** Effect of different co-surfactants on the emulsification efficiency of the selected oil phases with the selected surfactant Tween 80; data expressed as mean value ± S.D. (n = 3).

% Transmittance			
Oil	Labrasol	Transcutol-P	Propylene glycol
Cinnamon oil	100.08 ± 1.14	99.77 ± 0.61	100 ± 0.61
Clove oil	99.92 ± 0.48	100.16 ± 0.88	100.31 ± 0.7
Ethyl oleate	19.83 ± 1.33*	24.89 ± 0.67	29.55 ± 1.08*

*P < 0.05 when compared to Transcutol-P, **P < 0.001 when compared to Transcutol-P.

### The nature of obtained dispersions

It is essential to define the nature of the produced dispersions in order to use the appropriate methods for their characterization. Creating ternary or pseudo-ternary phase diagrams is one of the most frequently encountered points of confusion. Since the resulting microemulsion's phases are in equilibrium due to its thermodynamic stability, phase diagrams are created to examine its phase behavior. Nevertheless, it is incorrect to construct ternary phase diagrams using a nanoemulsion system since there is no equilibrium between the various phases due to the thermodynamic instability of nanoemulsions^32^. All of the obtained dispersions were unaffected by the forced temperature changes, indicating that they are nanoemulsions. Although temperature fluctuations can have a significant impact on the structure and droplet size of microemulsions, nanoemulsions are often robust systems that can withstand temperature changes without rapid destabilization^13^. As the temperature rises, microemulsions can transcend a phase barrier. Furthermore, clear dispersions were formed when Tween 80 and Transcutol-P were initially combined with clove oil before adding the aqueous phase. However, the emulsion did not develop when clove oil was introduced after Tween 80 and Transcutol-P had been dissolved in distilled water. Rather, two phases—a floating oil phase in the form of fine oil droplets and an aqueous phase—formed. Comparable outcomes were observed for systems based on ethyl oleate and cinnamon oil. As nanoemulsions are particularly sensitive to the order of mixing of their components, they can only be produced if surfactants are combined with the oil phase before adding the aqueous phase^14^. These results ascertain the nanoemulsion nature of the resulting dispersions. However, because of their thermodynamic stability, microemulsions remain precisely the same regardless of the order in which the various components are combined.

### Construction of pseudo-ternary phase diagrams

**Fig. 2:**
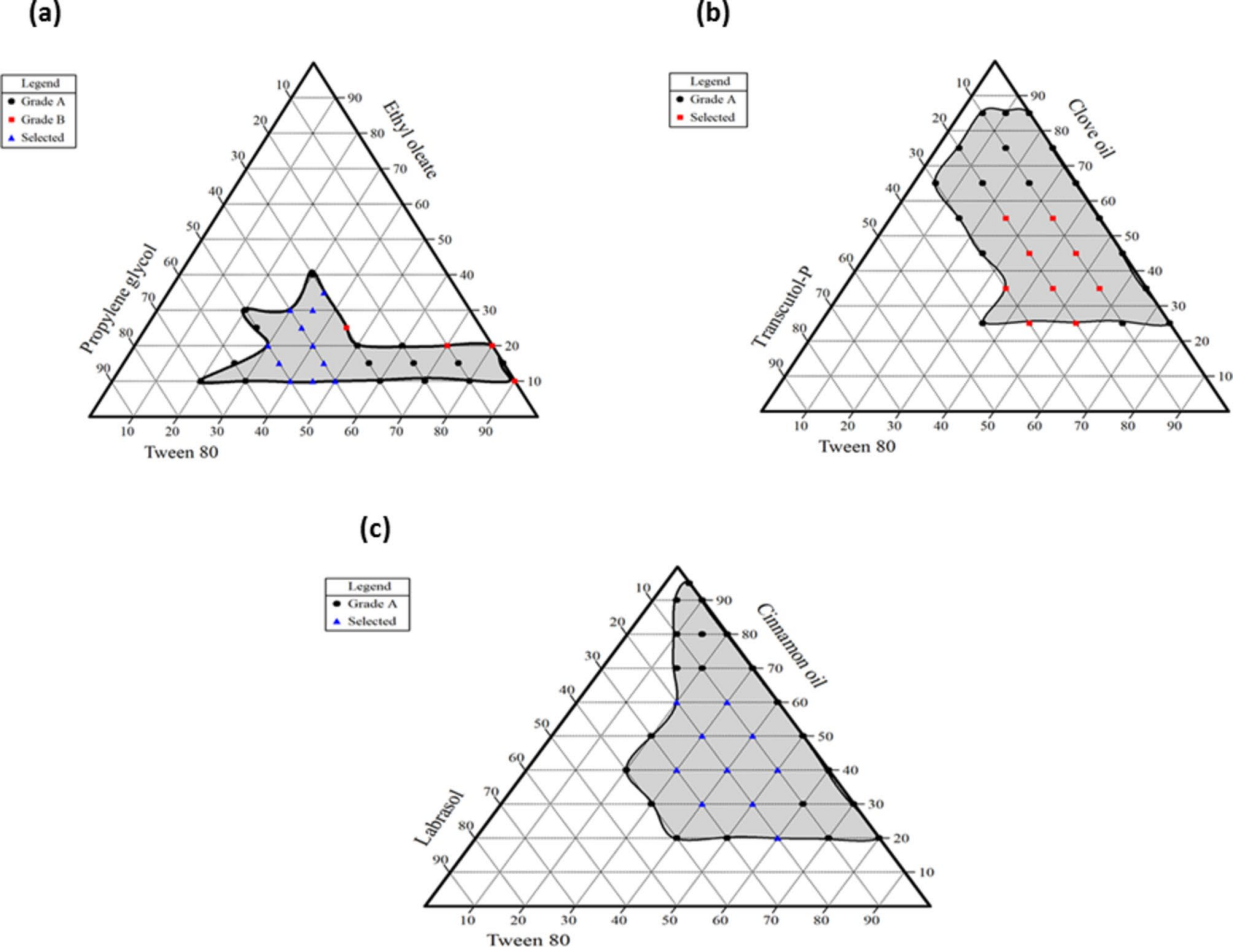
(**a**) pseudo-ternary phase diagram of an ethyl oleate-based system; (**b**) pseudo-ternary phase diagram of a clove oil-based system; (**c**) pseudo-ternary phase diagram of a cinnamon oil-based system. Grade A represents formulations with a percent transmittance higher than 95%; grade B represents formulations with a percent transmittance higher than 90%.

In order to select an appropriate concentration of the constituents, three pseudo-ternary phase diagrams were built to depict the self-emulsifying regions. A larger self-emulsifying area suggests that the system has better emulsification capabilities. The results inferred that increasing the surfactant concentration leads to the formation of small-sized globules with high transmittance values. Also, the addition of a co-solvent modifies the system's polarity and viscosity^33^. An excessive amount of co-surfactant will make the system less stable due to increasing its intrinsic aqueous solubility, resulting in larger droplets as the interfacial film expands^15^. This could be explained by increased water penetration into the oily droplets, leading to the ejection of oil droplets in the aqueous phase, which causes interfacial disruption^34^. As demonstrated in Fig. 2, essential oils showed larger nanoemulsion areas compared to ethyl oleate due to their higher polarity and lower hydrophobicity. Regarding essential oil, clear isotropic nanoemulsions could be produced with a minimum surfactant concentration of 5% w/w and a maximum oil concentration of 95% w/w. The high HLB value of the emulsifier system, which is matched with the HLB of the oil phase, can be utilized to explain the lower surfactant concentration used and the ease with which stable nanoemulsions were generated^35^. However, when the proportion of ethyl oleate reaches 40% w/w, microemulsions with preferable properties can hardly be formed. The results indicated that surfactant concentrations less than 20% w/w and co-surfactant concentrations higher than 70% w/w resulted in turbid emulsions. Since a formulation with a high surfactant ratio typically raises concerns about cell toxicity and gastrointestinal side effects, thirty formulations with lower surfactant concentrations and higher transmittance values were selected for further characterization (Supplementary Table S1).

### Stability of SNEDDS preconcentrates

Given that long-term storage causes drug precipitation and crystal formation, SNEDDS formulations need to be stable to avoid phase separation in the resulting dispersions^36^. To test this theory, several formulations were exposed to different stress conditions, and the effects on the stability of the resulting nanoemulsions were observed. With the exception of three formulations identified as CIN22, EO39, and EO44, none of the SNEDDS formulations showed any indications of turbidity, creaming, or phase separation (Supplementary Table S2).

### Robustness to dilution

Due to drug pH-dependent solubility, pH changes in the gastrointestinal tract (GIT) can cause drug precipitation in orally administered drug nanocarriers^36^. Therefore, the formulation should retain its stability against these pH changes. Moreover, the drug may precipitate at a higher dilution, which would delay the absorption process^37^. Simvastatin-loaded SNEDDS-based dispersions were therefore diluted up to 50, 100, and 1000 times with HCl (pH 1.2), phosphate buffer (pH 6.8), and distilled water to test their stability, and then they were visually observed for drug precipitation. The results demonstrated that no drug precipitation was observed in essential oil-based systems even after 1000 times dilution with either diluent, thus indicating formulation robustness to dilution. Such stability against dilutions indicates that the drug delivery systems are SNEDDS^38^. Because of the higher oil content, SNEDDS retains its solvent capacity in the aqueous phase. However, because of the lower oil concentration and increased concentration of hydrophilic surfactants and co-surfactants that promote phase separation, the dilution significantly affects the SMEDDS solvent capacity^39^. At pH 1.2 and 6.8, three formulations of the ethyl oleate-based system, designated EO15, EO23, and EO32, appeared milky white.

### Assessment of self-emulsification efficiency

Rapid and complete dispersion is required for SNEDDS formulations when diluted in water with mild agitation^40^. The ease of water penetration into the colloidal phases that form on the droplet surface determines the ease of emulsification. Essential oil-based systems passed the dispersibility test with a grade of A in all dispersing media. All of the formulations, irrespective of the kind of dispersing media, demonstrated a short emulsification time of less than one minute, demonstrating their great self-emulsification efficiency (Table 4). With regard to the ethyl oleate-based system, formulations EO 5, EO 7, EO 16, EO 24, and EO 40 produced an emulsion that quickly formed and had a slight bluish tinge in less than a minute; as a result, they were classified as grade A. EO 6, EO 15, EO 23, and EO 32 dispersions developed within three minutes and had an oily, milky appearance; as a result, they were graded as D. It could be observed that the self-emulsification time decreases with increasing co-solvent concentrations in the mixture, as the hydrophilic co-solvent makes the formulation more hydrophilic and facilitates easier water penetration. In the case of the ethyl oleate-based system, SNEDDS formulations with lower surfactant content produced the emulsion faster than other formulations. The longer emulsification time may be ascribed to the increased viscosity of formulations with high surfactant concentrations, which necessitate a higher shearing rate to form nanoemulsions.

**Table 4:** Self-emulsification time (second) of selected SNEDDS formulations in different dispersing media; data are expressed as the mean (n = 2).

Formulation code	Distilled water	Phosphate buffer (pH 6.8)	0.1 N HCl (pH 1.2)
CL 3	12.3 ± 2.4	15.7 ± 0.42	14.85 ± 1.63
CL4	9 ± 2.83	15.68 ± 0.18	12.35 ± 1.34
CL7	17.35 ± 0.92	29.55 ± 0.78	22.5 ± 1.56
CL8	10.75 ± 1.77	17.65 ± 0.92	16.5 ± 1.13
CL9	9.75 ± 1.06	8.75 ± 1.77	10.6 ± 0.85
CL10	13 ± 1.41	13.55 ± 1.06	18.75 ± 1.2
CL11	10.65 ± 1.63	12.2 ± 1.13	11.75 ± 1.77
CL14	11.6 ± 1.13	15.05 ± 0.35	12.3 ± 2.97
CL15	8.4 ± 0.99	11.25 ± 1.48	12.05 ± 1.48
CIN 3	19.5 ± 3.54	21.45 ± 2.19	22.2 ± 3.11
CIN 8	15.3 ± 1.27	19.5 ± 1.69	23.65 ± 0.92
Cin 9	14.85 ± 2.33	20.5 ± 0.71	19.5 ± 2.12
Cin 12	19.4 ± 2.26	19.9 ± 1.27	24.75 ± 1.06
Cin 13	16.1 ± 1.56	18.67 ± 0.47	14.6 ± 1.27
Cin 14	12.5 ± 1.84	15.73 ± 0.86	14.45 ± 2.62
Cin 17	15.15 ± 0.49	19.715 ± 0.59	18.41 ± 1.99
Cin 18	11.95 ± 0.78	14.05 ± 0.21	14.9 ± 1.13
Cin 21	14.25 ± 1.2	12.37 ± 0.52	10.6 ± 0.57
EO5	28 ± 0.71	22.55 ± 2.05	20.25 ± 0.64
EO6	88.8 ± 3.11	145 ± 2.83	134.5 ± 7.78
EO7	23.1 ± 0.85	29.7 ± 0.99	32.4 ± 1.41
EO15	151 ± 4.24	137 ± 5.66	170.5 ± 6.36
EO16	16.5 ± 0.57	24.35 ± 1.34	27.35 ± 0.49
EO23	143.5 ± 2.12	172 ± 4.24	151 ± 4.24
EO24	19.4 ± 1.69	28.05 ± 0.64	15.85 ± 0.35
EO32	158.5 ± 4.95	164.5 ± 3.54	141 ± 2.83
EO40	17.15 ± 0.64	19.7 ± 1.41	17.25 ± 0.78

### Characterization of selected SNEDDS formulations

#### Cloud point measurement

When assessing the stability of SNEDDS developed using non-ionic surfactants, determining the cloud point is essential^41^. The cloud point is the temperature at which an irreversible phase separation occurs; as a result, the dehydration of the non-ionic surfactants' polyethylene oxide moieties causes a clear formulation to turn turbid. The cloud point temperature of selected SNEDDS was found to be in the range of 60–95 °C (Table 5). Since they were over 37 °C, phase separation in the GIT was prevented. This led to the conclusion that a stable emulsion would be produced when these SNEDDS were given at body temperature^42^. The outcomes showed that the developed formulations don't need to be stored at a specific temperature because they are sufficiently stable.

**Table 5:** Cloud point, mean droplet size, PDI, and zeta potential of selected SNEDDS formulations; data are expressed as the mean value (n = 2).

Name	Mean droplet size (nm)	PDI	Zeta potential (mV)	Cloud point (°C)
CL 10	13.25 ± 0.03	0.157 ± 0.018	− 28.6 ± 10.8	84.5
CL 11	31.58 ± 1.05	0.212 ± 0.05	− 11.8 ± 2.54	79
CL 14	17.44 ± 0.229	0.282 ± 0.011	− 12.4 ± 6.08	82
CIN 17	15.34 ± 0.63	0.307 ± 0.013	− 9.90 ± 2.87	74
CIN 18	16.39 ± 0.166	0.178 ± 0.013	− 5.13 ± 1.05	60
EO 5	92.55 ± 0.395	0.297 ± 0.003	− 6.01 ± 3.73	88
EO 7	104.9 ± 0.79	0.303 ± 0.006	− 17.9 ± 0.82	94
EO 16	97.29 ± 0.83	0.340 ± 0.004	− 23.2 ± 2.91	75
EO 24	57.59 ± 0.185	0.573 ± 0.007	− 36.17 ± 2.05	72.5

#### Droplet size, polydispersity index and zeta potential determination

One of the most crucial factors affecting the in-vivo performance of SNEDDS is the droplet size of nanoemulsions^43^. Small-droplet-size SNEDDS formulations provide a large surface area for drug absorption and release^44^. Regarding the ethyl oleate-based system, the particle sizes of EO 5, EO 7, and EO 16 varied significantly (*P* < 0.001) (Table 5). The results showed that as the mass fraction of the surfactant increased (39.4%–49.4% w/w), the particle size of the microemulsion decreased from 104.9 ± 0.79 nm to 92.55 ± 0.39 nm. Ansari et al. and Kanwal et al. have reported the formation of smaller droplets from SNEDDS with a larger proportion of surfactant^45,46^. For clove oil-based systems, the particle sizes of CL 10, CL 11, and CL 14 varied significantly (*P* < 0.001). The results showed that by increasing the surfactant concentration (34.4%–44.4% w/w), the particle size decreased from 31.58 ± 1.05 nm to 13.25 ± 0.031 nm. However, by increasing the oil concentration (44.3%–54.3% w/w) and decreasing the co-solvent concentration (19.3%–9.3% w/w), the particle size decreased from 31.58 ± 1.05 nm to 17.44 ± 0.23 nm. Furthermore, after dilution with the aqueous phase, formulations demonstrated small PDI values, thus authenticating good uniformity of droplet size distribution. Zeta potential is a crucial parameter of SNEEDS droplets, as it plays a vital role in their physical stability^47^. Higher zeta potential causes higher repulsion, which prevents nanoemulsion droplets from coalescing^48^. For the chosen SNEDDS, the corresponding Zeta potential values varied between − 5.13 ± 1.05 mV and − 36.17 ± 2.05 mV (Table 5). The presence of free fatty acids may be the cause of the nanoemulsion droplets' negative charge^48^. In the case of the ethyl oleate-based system, however, the ester components of the oil phase might have played a role in the observed negative charge. The medium zeta potential values of EO5 (− 6.01 ± 3.73 mV) might result from the use of a larger concentration of non-ionic surfactant (Tween 80), which forms a covering layer around each system’s surface, providing steric stabilization of these systems^49^.

#### In-vitro drug release

**Fig. 3:**
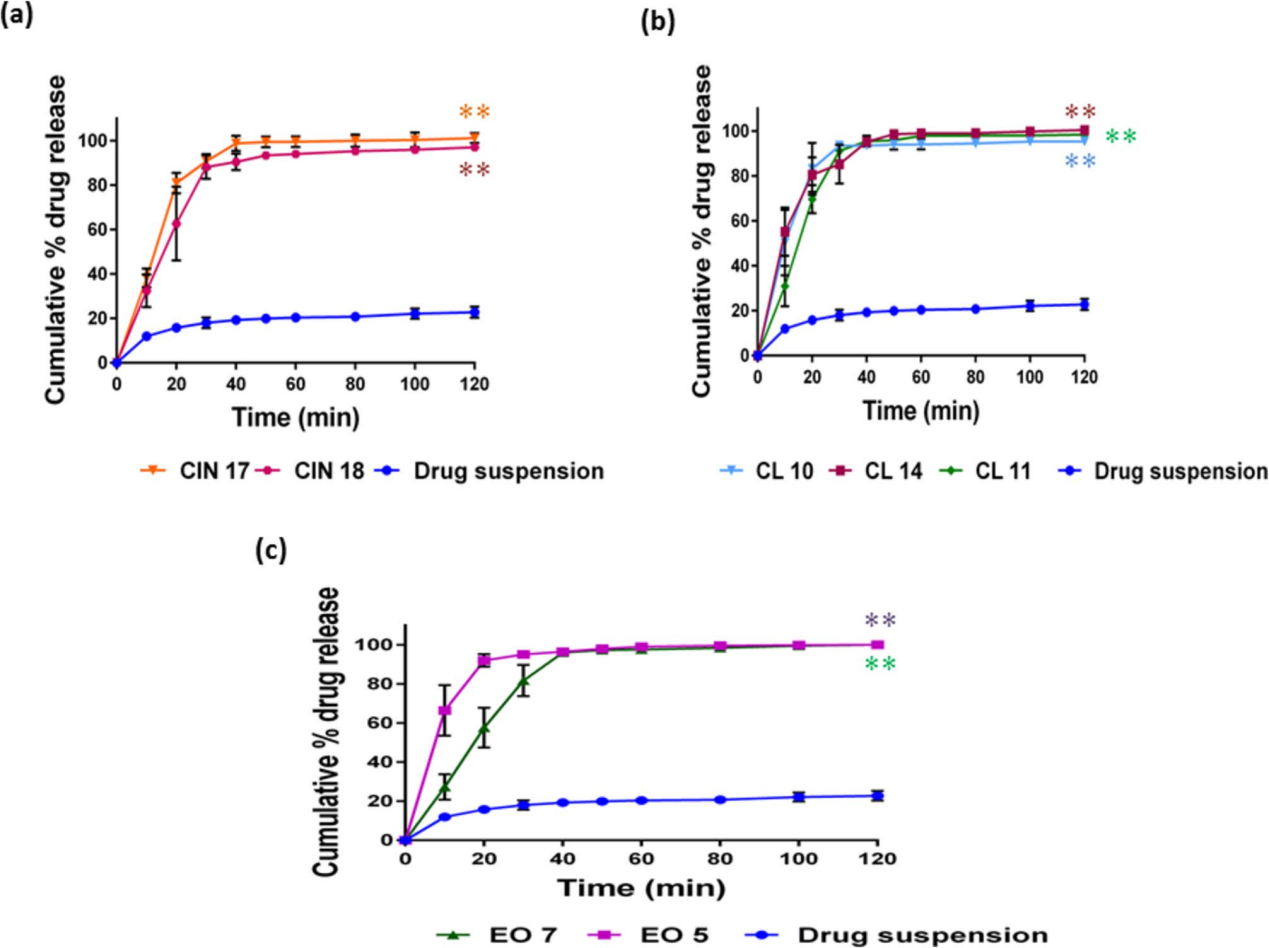
In vitro release of simvastatin in phosphate buffer, pH 7.4, data presented as mean ± SD, n = 3. (**a**) cinnamon oil-based system; (**b**) clove oil-based system; and (**c**) ethyl oleate-based system. *P < 0.05 when compared to drug suspension; **P < 0.001 when compared to drug suspension.

**Fig. 4:**
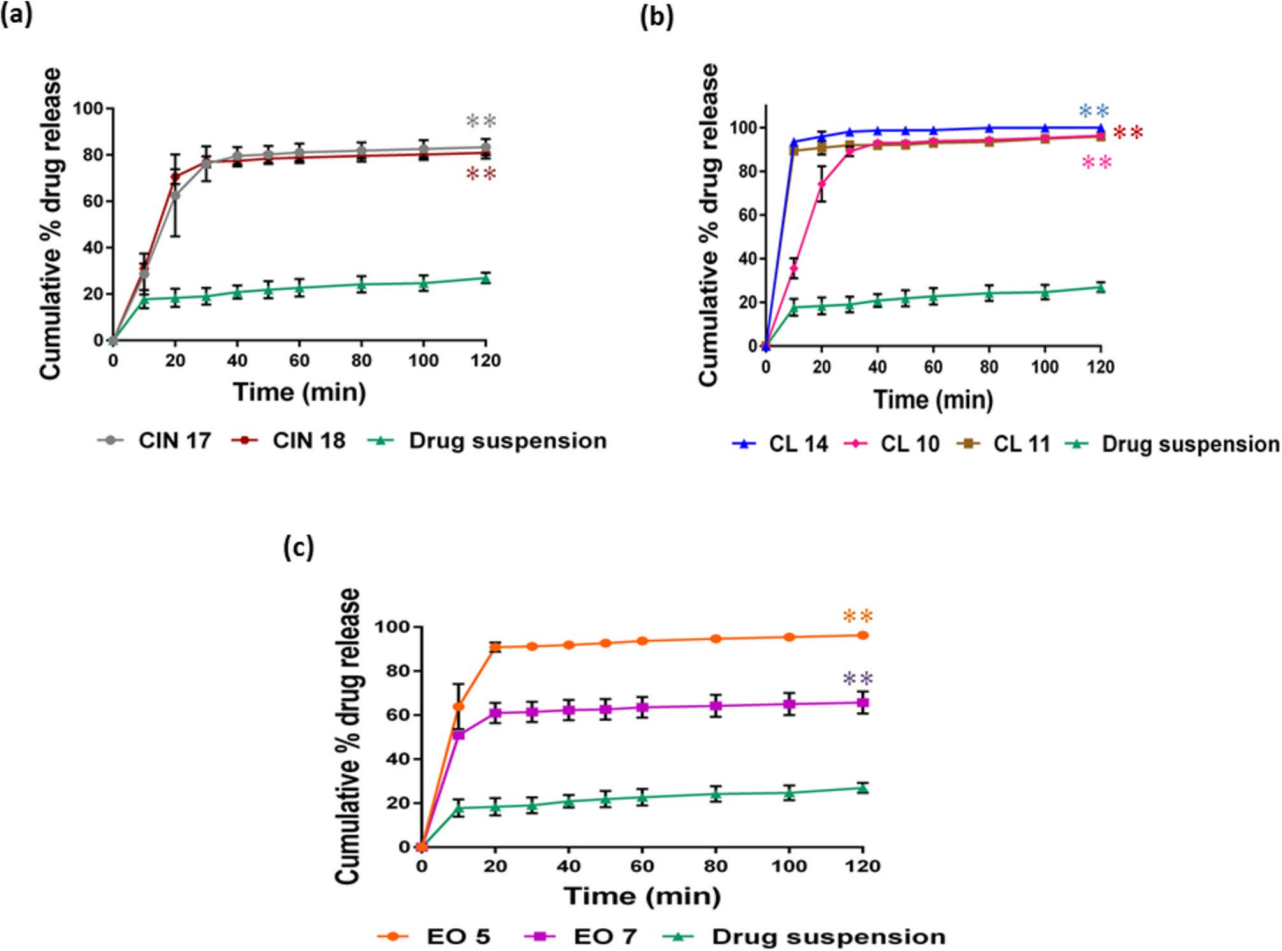
In vitro release of simvastatin in 0.1 N HCl; data presented as mean ± SD, n = 3. (**a**) cinnamon oil-based system; (**b**) clove oil-based system; and (**c**) ethyl oleate-based system. *P < 0.05 when compared to drug suspension; **P < 0.001 when compared to drug suspension.

The percent release of simvastatin from all formulations in the initial 40 min was more than 90% in comparison to the drug suspension, which showed 22.8% release in phosphate buffer even after 2 h (Fig. 3). The results indicated that drug release from all the screened formulations was very highly significant compared to that from drug suspension (*P* < 0.0001), thus suggesting a higher bioavailability potential of the drug. This could be explained by the larger surface area for drug release caused by the increased availability of dissolved simvastatin from the nanosized droplets. Maximum drug release was observed with formulations CL 11, CL 14, CIN 17, EO 5, and EO 7, with non-significant differences among them. This may be because of their small mean droplet size and the formulations' optimal oil and surfactant content. The release of drugs from CL 10 and CIN 18 was significantly lower than from CL 11, CL 14, CIN 17, EO 5, and EO 7 (*p* < 0.05). The reduced drug release in the CL-10 formulation could have been caused by the higher amount of surfactants, which increased the probability of surfactant migration during dispersion into the surrounding aqueous medium. As a result of this process, micelles are formed, which trap the free drug and create hindrances to its release. In general, the quantitative drug release from developed nanoemulsions is droplet size-dependent. This suggests that the higher interfacial area of small droplet nanoemulsions facilitates faster drug release^50^. Analogous in-vitro dissolution profiles from ethyl oleate-based formulations and clove oil-based formulations show that the lipidic chain length does not appear to have a major impact on the drug release from the SNEDDS. The drug release patterns in phosphate buffer and acidic medium were found to be approximately similar to each other in EO 5, CL 10, CL 11, and CL 14 formulations. As shown in Fig. 4, drug release from the above-mentioned formulations was significantly higher than from CIN 17, CIN 18, and EO 7. This could be explained by the smaller particle size of EO 5 compared to EO 7. Since both EO 5 and EO 7 have low oil contents, the hydrophilic surfactants and co-solvents are the primary solubilizing components. Hence, the higher co-solvent content in EO 7 resulted in a higher precipitation tendency. The three formulations, CL 11, CL 14, and EO 5, were quickly dissolved regardless of the fluid conditions. A variety of mathematical models were used to assess the simvastatin release mechanism of the chosen SNEDDS in phosphate buffer (pH 7.4) and 0.1 N HCl (pH 1.2). The first-order model that provided the best fit for the drug release from the different formulations was found to be the primary release mechanism^51^.

### Further characterization of the selected formulations

Both CL 11 and CL 14 formulations combined the larger oil content (55% w/w) for CL 14 and (45% w/w) for CL 11, which is advantageous for SNEDDS formulations with small droplets. The cloud point, zeta potential, and release pattern of both formulations were comparable. However, CL 14 showed a smaller droplet size and PDI; thus, it was selected for further evaluation. EO 5 was selected for further characterization of the ethyl oleate-based system because it demonstrated higher drug release in an acidic medium and a smaller particle size than EO 7.

#### Transmission electron microscopy (TEM)

**Fig. 5:**
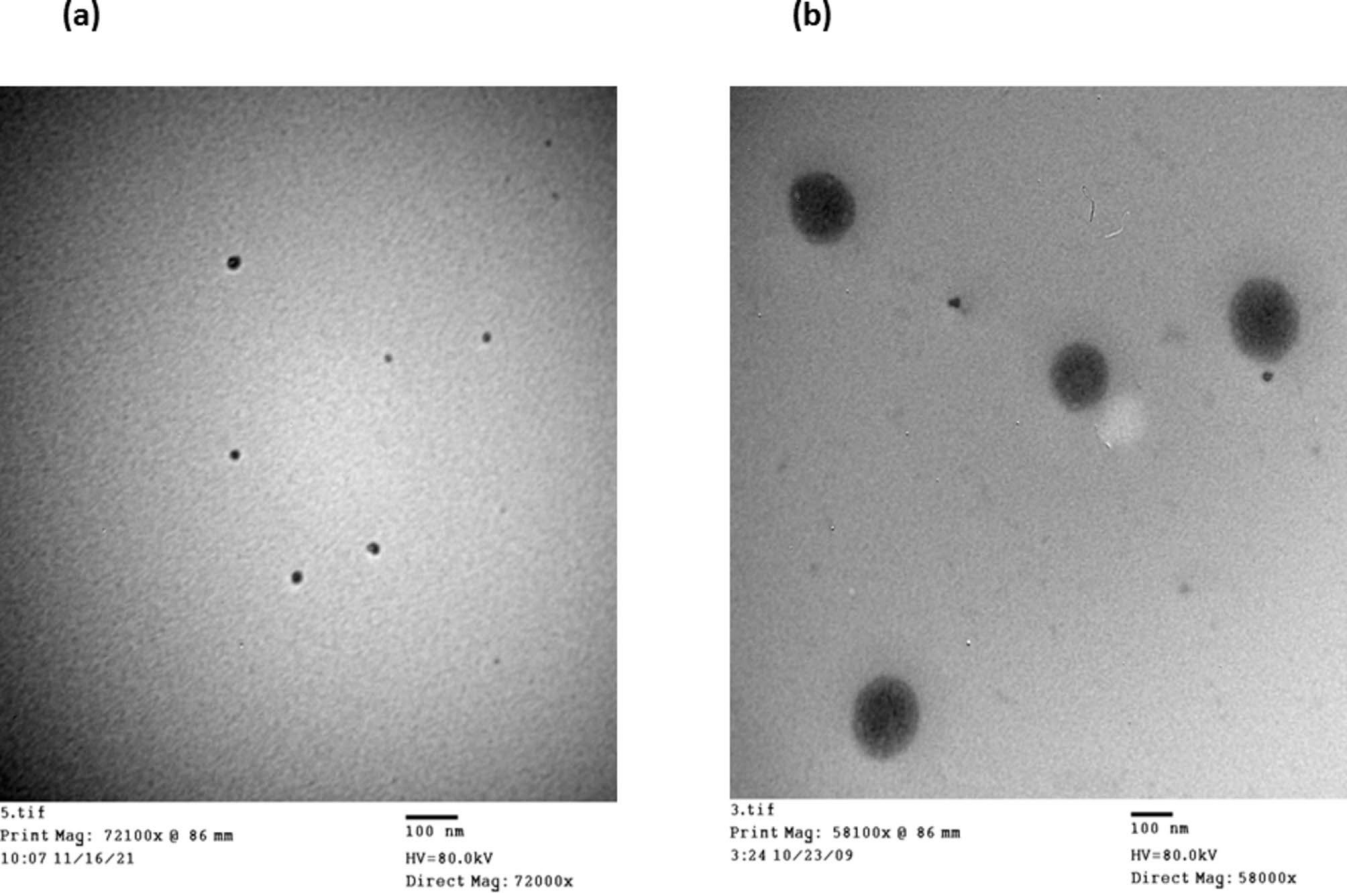
(**a**) TEM photograph of EO 5 formulation; (**b**) TEM photograph of CL 14 formulation.

The photographs depicted in Fig. 5 showed that all droplets possessed a spherical shape with no signs of aggregation up to 24 h post-dilution. Because microemulsions can adopt various configurations, the spherical shape of the formulations further supports their nanoemulsion nature. Furthermore, the evenly distributed globules and lack of coalescence indicated that the resulting nanoemulsion had good physical stability.

#### Stability of the simvastatin loaded-SNEDDS formulations

Both CL 14 and EO 5 were found to be stable for three months at room temperature (25 °C). There was no significant change in drug content, mean droplet size, or zeta potential of the resultant nanoemulsions (Tables 6 and 7). In addition, there was no significant change in appearance, clarity, or self-emulsification time after dilution.

**Table 6:** Evaluation data of CL 14 subjected to a stability study; data are expressed as the mean value (n = 3).

Time (month)	% T	SET (second)	Mean droplet size (nm)	Zeta potential (mV)	Drug content (%)
0	99.85 ± 1.53	10.5 ± 0.92	19.30 ± 2.05	− 17.7 ± 2.19	100.42 ± 5.88
1	100.38 ± 0.82	11.53 ± 1.33	18.68 ± 0.588	− 16.5 ± 4.03	100.91 ± 2.47
2	100.62 ± 1.28	10.93 ± 1.7	17.46 ± 1.38	− 13.7 ± 5.64	99.46 ± 0.56
3	99.7 ± 0.93	10.77 ± 1.89	22.37 ± 0.979	− 17.3 ± 1.21	99.95 ± 1.41

**Table 7:** Evaluation data of EO 5 subjected to a stability study; data are expressed as the mean value (n = 3).

Time (month)	% T	SET (second)	Mean droplet size (nm)	Zeta potential (mV)	Drug content (%)
0	98.78 ± 0.57	27.97 ± 0.85	100.2 ± 1.026	− 13.8 ± 1.59	99.13 ± 1.92
1	97.73 ± 0.81	27.5 ± 1.23	103.4 ± 0.17	− 11.8 ± 0.058	99.46 ± 2.94
2	98.33 ± 0.69	28.4 ± 1.18	97.02 ± 2.83	− 17.8 ± 0.794	101.06 ± 4.19
3	97.49 ± 0.45	27.8 ± 1.81	81.53 ± 0.71	− 14.8 ± 0.52	99.78 ± 2.78

#### Effects of simvastatin-loaded SNEDDS on hyperlipidemia

**Fig. 6:**
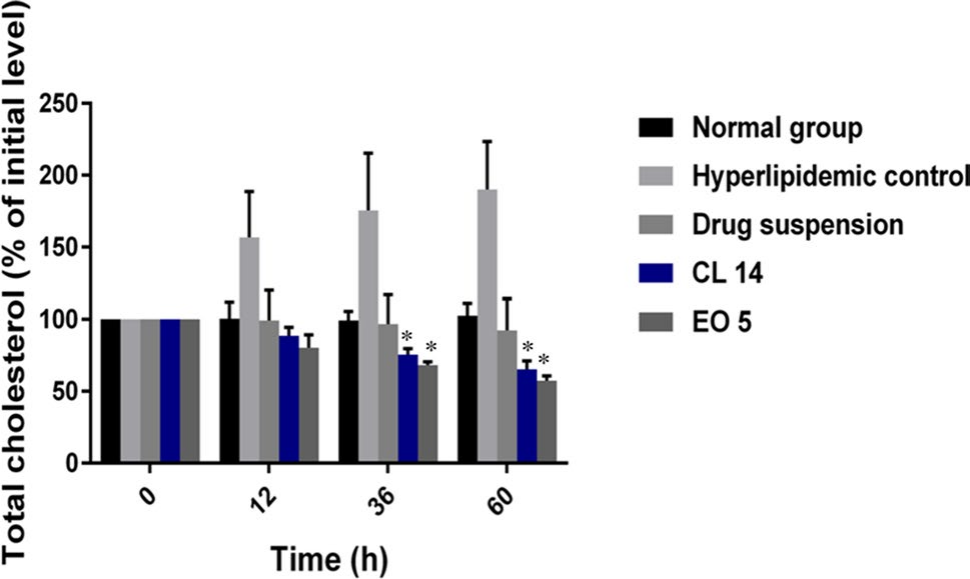
Changes in TC level in rats after oral administration of pure simvastatin suspension and simvastatin-loaded SNEDDS over three days. Each value is expressed as the mean ± standard deviation (n = 6). *P < 0.05 when compared to the free drug; **P < 0.001 when compared to the free drug.

**Fig. 7:**
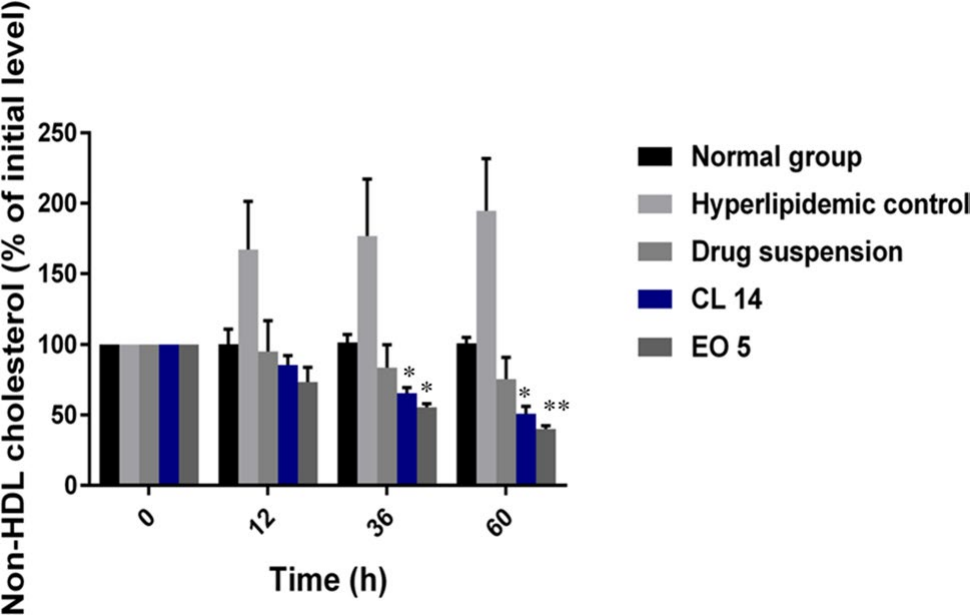
Changes in non-HDL levels in rats after oral administration of pure simvastatin suspension and simvastatin-loaded SNEDDS over three days. Each value is expressed as the mean ± standard deviation (n = 6). *P < 0.05 when compared to the free drug; **P < 0.001 when compared to the free drug.

By monitoring serum lipid profiles for 60 h, the anti-hyperlipidemic effect of simvastatin-loaded SNEDDS in a poloxamer-induced hyperlipidemic rat model was assessed. Poloxamer 407 offers an attractive method of hyperlipidemia induction due to its rapid onset, lack of toxicity, and ability to produce intense hyperlipidemia within a few hours^52^. Poloxamer 407 acts by various mechanisms, including the inhibition of lipoprotein lipase, which is responsible for the hydrolysis of triglycerides, thus interfering with the lipid metabolism, and indirect stimulation of HMG-CoA reductase, which is involved in cholesterol biosynthesis^53,54^. In all groups, the injection of poloxamer 407 resulted in an increase in serum total cholesterol and non-HDL-C levels. However, there was no significant change in the lipid profile in the normal group that received normal saline injections. These results indicated a successful induction of hyperlipidemia in rats after 12 h of poloxamer injection. Oral administration of simvastatin-loaded SNEDDS and simvastatin suspension was started after 12 h and continued for 60 h. The effects of simvastatin-loaded SNEDDS and simvastatin suspension treatment on total cholesterol and non-HDL-C profiles after 60 h are presented in Figs. 6 and 7. Both the selected SNEDDS formulations (CL 14 and EO 5) significantly modified the plasma TC and non-HDL-C levels as compared to the control and simvastatin suspensions. The lipid profile of the hyperlipidemic control group showed a ceaseless rise in total cholesterol and non-HDL-C levels. CL 14 and EO 5 were able to significantly reduce the plasma TC levels by 34.8% and 42.54%, respectively, as compared to drug suspension. Both the selected formulations were able to markedly reduce the plasma non-HDL-C levels by 49.1% and 59.2% versus drug suspension. The better performance of the selected SNEDDS formulations could be attributed to increased solubility of the drug, leading to fast and complete absorption of the drug^55^. The marked improvement in plasma lipid levels observed with EO 5 may be attributed to ethyl oleate, since it has been reported to circumvent the hepatic first-pass effect, hence facilitating drug transportation via the lymphatic system and enhancing drug oral bioavailability^56^. The intraluminal processing of the triglycerides depends on the chain length of the lipids^57^. Long-chain triglycerides were shown to have improved intestinal absorption and solubilization capacity, which augmented lymphatic transport. This effect results in decreased hepatic first-pass metabolism and increased bioavailability of oral drugs.

### Pharmacokinetic study

**Fig. 8:**
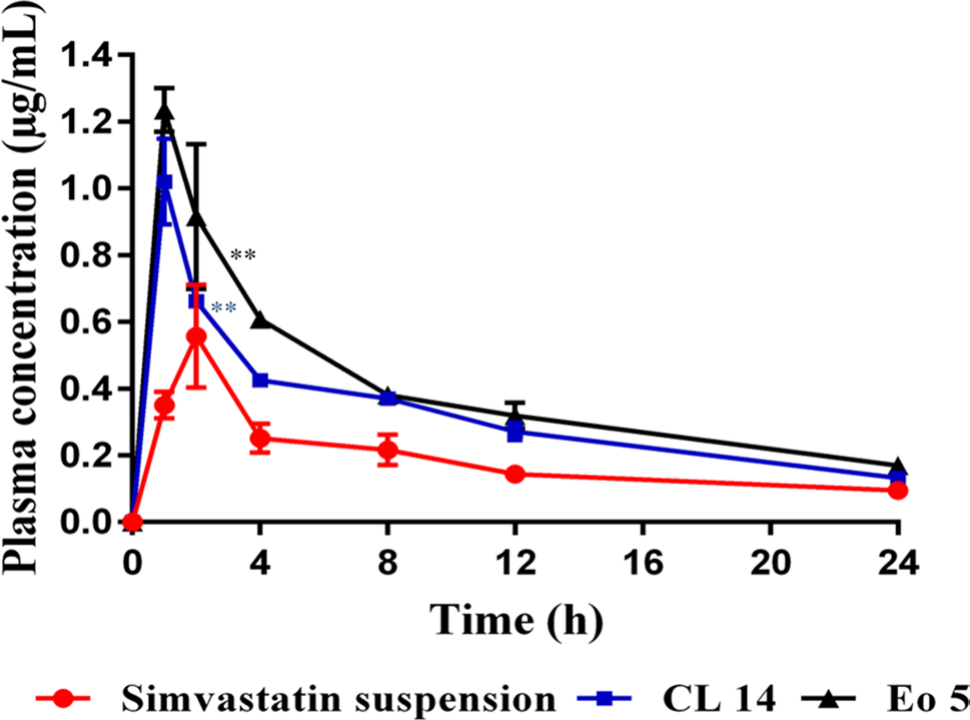
Plasma concentration and time profiles of simvastatin after a single oral dose (40 mg/kg) of simvastatin-loaded SNEDDS (CL 14 and EO 5) and simvastatin suspension. Values are presented as means ± SD (n = 3). *P < 0.05 when compared to the free drug; **P < 0.001 when compared to the free drug.

The pharmacokinetic behavior of simvastatin-loaded SNEDDS (EO 5 and CL 14) formulations following oral administration to rats was evaluated and compared to that of simvastatin suspension. With some modifications, a previously reported HPLC method was used to assess simvastatin plasma concentrations^23^. Simvastatin was completely separated as a sharp peak at a retention time of 8.2 min without any interfering peaks. The plasma concentration versus time curves were illustrated in Fig. 8, and the estimated pharmacokinetic parameters were summarized in Table 8. The simvastatin plasma concentrations were remarkably higher after administration of simvastatin-loaded SNEDDS, as compared to those observed for simvastatin suspension. The peak plasma concentrations of simvastatin from CL 14 and EO 5 were 1.02 ± 0.13 µg/mL and 1.24 ± 0.07 µg/mL, which were improved by 1.83 fold and 2.22 fold compared with simvastatin suspension, respectively. Additionally, the C_max_ of simvastatin was 2 h after oral administration of simvastatin suspension but shortened to 1 h after administration of simvastatin-loaded SNEDDS. The enhancement of C_max_ and shortening of t_max_ were a consequence of the significantly enhanced absorption of simvastatin following its formulation as a SNEEDS. In addition, the AUC_0-∞_ value of simvastatin in rats treated with simvastatin suspension (6.53 ± 0.18 µg/ml*h) was significantly enhanced following its formulation as a SNEEDS (*p* ˂ 0.001). The values of AUC_0-∞_ of SNEDDS formulations CL 14 and EO 5 were 9.87 ± 0.91 µg/ml*h and 12.72 ± 0.38 µg/ml*h, which were improved by 1.51 fold and 1.95 fold compared with simvastatin suspension, respectively. Similar enhancements in systemic absorption of the same drug were previously reported using other drug delivery systems, such as nanostructured lipid carriers^4^. After oral administration, simvastatin-loaded SNEDDS have an increased absorption extent and a higher bioavailability, as indicated by their larger AUC and C_max_. Several mechanisms, such as the drug's presentation in a solubilized state, a greater interfacial area for absorption, and improved dissolution in the presence of surfactants, may account for the superior oral bioavailability of selected simvastatin-loaded SNEDDS formulations^58^. Additionally, SNEDDS improves drug oral bioavailability by avoiding P-glycoprotein efflux and cytochrome P-450-mediated drug metabolism and by improving transportation via lymphatic pathways^59^. It is believed that because of its small droplet size, SNEDDS is absorbed from the small intestines and reaches the systemic circulation via the lymphatic system^60^. There is debate over the impact of different lipid types, such as those based on medium-chain fatty acids (MCFA) or long-chain fatty acids (LCFA), on increasing the bioavailability of medications. The findings showed that EO 5, a formulation based on LCFA, showed better bioavailability. Long-chain fatty acids, with the presence of high surfactant components (Tween 80), can circumvent the portal circulation and enter the intestinal lymph, which may account for EO 5's higher bioavailability compared to CL 14^19^. Hence, SNEDDS could be considered a multifunctional delivery system for enhancing the poor oral bioavailability of simvastatin by various mechanisms simultaneously.

**Table 8:** Pharmacokinetic parameters after oral administration of simvastatin-loaded SNEDDS and simvastatin suspension (equivalent to 40 mg/kg of simvastatin) to rats; data are expressed as the mean value (n = 3).

Pharmacokinetic parameters	Drug suspension	SNEDDS (CL 14)	SNEDDS (EO5)
Ke (1/h)	0.05 ± 0.01	0.06 ± 0.003*	0.05 ± 0.006
t_½_(h)	14.52 ± 2.96	11.16 ± 0.58*	13.05 ± 1.33
T_max_ (h)	2	1	1
C_max_ (µg/mL)	0.5568 ± 0.1545	1.02 ± 0.1288*	1.235 ± 0.065**
AUC_0-24_ (µg/ml*h)	4.532 ± 0.584	7.7349 ± 0.518**	9.5314 ± 0.543**
AUC_0-∞_ (µg/ml*h)	6.533 ± 0.176	9.865 ± 0.905**	12.7239 ± 0.384**
AUMC_0-∞_ (µg/ml*h^2^)	130.366 ± 24.375	150.607 ± 22.89	215.679 ± 13.692*
MRT_0-∞_ (h)	20.012 ± 4.27	15.224 ± 0.92*	16.967 ± 1.305
F (%)	100	151	194.7

*p < 0.05 when compared to the free drug; **p < 0.001 when compared to the free drug.

## Conclusion

The primary goal of the current investigation was to overcome simvastatin's limitations, such as its limited solubility and poor bioavailability. Through the development of SNEDDS based on various types of oil, including ethyl oleate, cinnamon oil, and clove oil, the physicochemical attributes of simvastatin were successfully improved. A comprehensive analysis was conducted on two formulations: EO 5, which is comprised of ethyl oleate, tween 80, and propylene glycol at a weight ratio of 9.3:49.4:39.3, and CL 14, which is composed of clove oil, tween 80, and transcutol-P at a ratio of 54.3:34.4:9.3. They displayed desirable characteristics such as stability, small droplet size, low PDI values, and moderate zeta potential. This was reflected in the superior in vitro release profile of simvastatin from both formulations compared to simvastatin suspension. Furthermore, simvastatin's anti-hyperlipidemic effect and bioavailability were markedly enhanced by both formulations. The developed SNEDDS (EO 5 and CL 14) increased the oral bioavailability of simvastatin in rats by 1.95 and 1.51 fold, respectively, in comparison to the drug suspension. The higher bioavailability of EO 5 could potentially be attributed to the presence of long-chain fatty acids, which circumvent the portal circulation and enter the intestinal lymph.

## Supplementary Information

Supplementary Tables.

## Data Availability

The datasets analyzed during the current study are available from the corresponding author on reasonable request.

## Abbreviations

BCS: Biopharmaceutical classification system
CYP3A: Cytochrome P3A
CMC: Sodium carboxymethyl cellulose
DLS: Dynamic light scattering
HCl: Hydrochloric acid
HDL: High-density lipoproteins
HLB: Hydrophilic-lipophilic balance
HMG-CoA: 3-Hydroxy-3-methyl-glutaryl-coenzyme A
LCFA: Long-chain fatty acids
MCFA: Medium-chain fatty acids
Non HDL-C: Non-high density lipoproteins-cholesterol
PDI: Polydispersity index
P-gp: P-glycoprotein
SD: Standard deviation
SET: Self-emulsification time
SIM: Simvastatin
SMEDDS: Self-microemulsifying drug delivery systems
SNEDDS: Self-nanoemulsifying drug delivery systems
TEM: Transmission electron microscopy
TC: Total cholesterol
UV–VIS: Ultraviolet–visible
