# Supplementary material for: Impact of oil type on the development and oral bioavailability of self-nanoemulsifying drug delivery systems containing simvastatin
Source: Sci Rep. 2024 Sep 29;14:22584. doi: 10.1038/s41598-024-71980-5 (PMC11439918; doi:10.1038/s41598-024-71980-5)
Supplement: Supplementary file 1 — Supplementary Tables. [file 41598_2024_71980_MOESM1_ESM.pdf]

Title:

Impact of oil type on the development and oral bioavailability of self-nanoemulsifying drug delivery systems containing simvastatin

Authors:

Aya Hamdy[1*], Mahmoud El-Badry[2], M. Fathy[2], Ahmed M. El-Sayed[2]

Affilations:

[1]Assistant lecturer, Department of Pharmaceutics, Faculty of Pharmacy, Assiut University, Assiut, 71526, Egypt.

[2]Emeritus Professor, Department of Pharmaceutics, Faculty of Pharmacy, Assiut University, Assiut, 71526, Egypt.

Emails:

elbadry@aun.edu.eg

mohamed.ibrahim9@pharm.aun.edu.eg

ahmed.mostafa70020@yahoo.com

Author for correspondence:

Aya Hamdy, Department of Pharmaceutics, Faculty of Pharmacy, Assiut University, Assiut, 71526, Egypt.

E-mail:

ayatallahhamdy@pharm.aun.edu.eg

aya.hamdy31@yahoo.com

Telephone: +201124739283

Address: Egypt, Assiut, El fath, 71785.

Supplementary Table S1. Percentage transmittance of selected SNEDDS formulations.

**Formulation**	**% Transmittance**	**Formulation**	**% Transmittance**
Cin 3	100.46 ± 0.84	EO5	97.43 ± 1.23
Cin 8	100.31 ± 0.96	EO6	98.71 ± 0.8
Cin 9	100.77 ± 0.82	EO7	99.16 ± 0.57
Cin 12	101.78 ± 0.97	EO15	98.48 ± 0.92
Cin 13	101.7 ± 0.36	EO16	100.39 ± 0.58
Cin 14	101.39 ± 0.93	EO23	98.41 ± 1.36
Cin 17	100 ± 0.23	EO24	99.31 ± 0.69
Cin 18	99.85 ± 0.48	EO32	98.55 ± 0.8
Cin 21	99.85 ± 0.81	EO39	99.08 ± 0.46
Cin 22	99.46 ± 0.48	EO40	99.69 ± 0.58
Cl 3	100 ± 1.28	EO44	96.98 ± 1.23
Cl 4	100.16 ± 0.93	Cl 10	100.23 ± 0.61
Cl 7	99.77 ± 0.69	Cl 11	99.77 ± 0.998
Cl 8	99.85 ± 0.48	Cl 14	99.93 ± 1.63
Cl 9	99.7 ± 0.93	Cl 15	100.24 ± 1.88

Supplementary Table S2. Composition and thermodynamic stability test results of selected SNEDDS formulations.

Name	Oil (%)	Surfactant (%)	Co-surfactant (%)	Centrifugation	Heating-cooling cycle	Freeze-thaw cycle
**Cin 3**	19.3 Cinnamon oil	59.4 Tween 80	19.3 Labrasol	Stable	Stable	Stable
**Cin 8**	29.3 Cinnamon oil	49.4 Tween 80	19.3 Labrasol	Stable	Stable	Stable
**Cin 9**	29.3 Cinnamon oil	39.4 Tween 80	29.3 Labrasol	Stable	Stable	Stable
**Cin 12**	39.3 Cinnamon oil	49.4 Tween 80	9.3 Labrasol	Stable	Stable	Stable
**Cin 13**	39.3 Cinnamon oil	39.4 Tween 80	19.3 Labrasol	Stable	Stable	Stable
**Cin 14**	39.3 Cinnamon oil	29.4 Tween 80	29.3 Labrasol	Stable	Stable	Stable
**Cin 17**	49.3 Cinnamon oil	39.4 Tween 80	9.3 Labrasol	Stable	Stable	Stable
**Cin 18**	49.3 Cinnamon oil	29.4 Tween 80	19.3 Labrasol	Stable	Stable	Stable
**Cin 21**	59.3 Cinnamon oil	29.4 Tween 80	9.3 Labrasol	Stable	Stable	Stable
**Cin 22**	59.3 Cinnamon oil	19.4 Tween 80	19.3 Labrasol	Stable	Instable	Instable
**Cl 3**	24.3 Clove oil	54.4 Tween 80	19.3 Transcutol-P	Stable	Stable	Stable
**Cl 4**	24.3 Clove oil	44.4 Tween 80	29.3 Transcutol-P	Stable	Stable	Stable
**Cl 7**	34.3 Clove oil	54.4 Tween 80	9.3 Transcutol-P	Stable	Stable	Stable
**Cl 8**	34.3 Clove oil	44.4 Tween 80	19.3 Transcutol-P	Stable	Stable	Stable
**Cl 9**	34.3 Clove oil	34.4 Tween 80	29.3 Transcutol-P	Stable	Stable	Stable
**Cl 10**	44.3 Clove oil	44.4 Tween 80	9.3 Transcutol-P	Stable	Stable	Stable

Name	Oil (%)	Surfactant (%)	Co-surfactant (%)	Centrifugation	Heating-cooling cycle	Freeze-thaw cycle
**Cl 11**	44.3 Clove oil	34.4 Tween 80	19.3 Transcutol-P	Stable	Stable	Stable
**Cl 14**	54.3 Clove oil	34.4 Tween 80	9.3 Transcutol-P	Stable	Stable	Stable
**Cl 15**	54.3 Clove oil	24.4 Tween 80	19.3 Transcutol-P	Stable	Stable	Stable
**EO5**	9.3 Ethyl oleate	49.4 Tween 80	39.3 Propylene glycol	Stable	Stable	Stable
**EO6**	9.3 Ethyl oleate	44.4 Tween 80	44.4 Propylene glycol	Stable	Stable	Stable
**EO7**	9.3 Ethyl oleate	39.4 Tween 80	49.3 Propylene glycol	Stable	Stable	Stable
**EO15**	14.3 Ethyl oleate	44.4 Tween 80	39.3 Propylene glycol	Stable	Stable	Stable
**EO16**	14.3 Ethyl oleate	34.4 Tween 80	49.3 Propylene glycol	Stable	Stable	Stable
**EO23**	19.3 Ethyl oleate	39.4 Tween 80	39.3 Propylene glycol	Stable	Stable	Stable
**EO24**	19.3 Ethyl oleate	29.4 Tween 80	49.3 Propylene glycol	Stable	Stable	Stable
**EO32**	24.3 Ethyl oleate	34.4 Tween 80	39.3 Propylene glycol	Stable	Stable	Stable
**EO39**	29.3 Ethyl oleate	34.4 Tween 80	34.3 Propylene glycol	Instable	Instable	Instable
**EO40**	29.3 Ethyl oleate	29.4 Tween 80	39.3 Propylene glycol	Stable	Stable	Stable
**EO44**	34.3 Ethyl oleate	34.4 Tween 80	29.3 Propylene glycol	Instable	Instable	Instable
